# Comparing Traditional and Digital Cognitive Assessments in an Online Dementia Risk Reduction Program: Results from the CAN‐THUMBS‐UP Brain Health Support Program

**DOI:** 10.1002/alz70857_106063

**Published:** 2025-12-25

**Authors:** Paul W.H. Brewster, Diane M. Jacobs, Nicole D. Anderson, John R. Best, Manuel Montero‐Odasso, Sylvie Belleville, Haakon B. Nygaard, Howard H. Feldman, Howard Chertkow, CAN‐THUMBS‐UP Study Group

**Affiliations:** ^1^ University of Victoria, Victoria, BC, Canada; ^2^ Alzheimer's Disease Cooperative Study, University of California San Diego, La Jolla, CA, USA; ^3^ Rotman Research Institute, Baycrest Academy for Research and Education, Toronto, ON, Canada; ^4^ Kimel Family Centre for Brain Health and Wellness, Toronto, ON, Canada; ^5^ Simon Fraser University, Vancouver, BC, Canada; ^6^ Parkwood Institute, London, ON, Canada; ^7^ Université de Montréal, Montréal, QC, Canada; ^8^ UBC Hospital Clinic for Alzheimer Disease and Related Disorders, Vancouver, BC, Canada; ^9^ Alzheimer's Disease Cooperative Study, University of California San Diego, La Jolla, CA, USA; ^10^ Division of Neurology, Department of Medicine, University of Toronto, Toronto, ON, Canada; ^11^ Canadian Consortium on Neurodegeneration in Aging (CCNA), Montreal, QC, Canada; ^12^ Kimel Family Centre for Brain Health and Wellness and Anne & Allan Bank Centre for Clinical Research Trials, Baycrest Health Sciences, Toronto, ON, Canada

## Abstract

**Background:**

The CAN‐THUMBS‐UP Brain Health Support Program (BHP; NCT05347966), a year‐long web‐based dementia risk reduction study, included two self‐administered digital cognitive assessments as exploratory outcomes. Cogniciti's Brain Health Assessment (BHA), a web‐based screening battery, was completed every 6‐months. MyCogHealth, a smartphone‐based ecological momentary assessment, was deployed in 7‐day “bursts” every 3‐months. Here, we compare these novel assessment approaches to a traditional neuropsychological battery (NTB) delivered remotely at study baseline and endpoint.

**Method:**

Global composites from the NTB, BHA, and MyCogHealth were analyzed using mixed models for repeated measures (MMRM) covarying for age, sex, education, and cognitive status. Time was modeled categorically. A lifestyle composite, derived from seven questionnaires completed in three‐month intervals, was modeled as a time‐varying covariate. We used structural equation modeling of BHA and MyCogHealth composites to investigate coupled changes in cognition and lifestyle (dual‐growth models).

**Result:**

MMRM analyses revealed significant effects of age (β=‐0.307 to ‐0.142, *p* <0.001), education (β=0.066 to 0.095, *p* <0.05), and cognitive status (β=‐0.292 to ‐0.176, *p* <0.001) on baseline cognition across measurement approaches. There were no consistent effects of time on cognition, or of covariates on change over time. The lifestyle composite was not associated with cognition.

Dual growth models confirmed baseline associations of MyCogHealth and BHA with age, education, and cognitive status (ps<0.01). Longitudinally, the BHA remained stable, whereas MyCogHealth improved (β=0.342, *p* <0.001) with quadratic attenuation (β=‐0.049, *p* <0.001). Education was marginally associated with improvement on MyCogHealth (β=0.029, *p* = 0.047). Age negatively predicted MyCogHealth intercept (β=‐0.350, *p* <0.001), but also predicted more improvement (β=0.046, *p* = 0.009) and faster attenuation over time (β=‐0.009, *p* = 0.007). Males trended less improvement (β=‐0.047, *p* = 0.09) and slower attenuation (β=0.014, *p* <0.05). The **lifestyle composite intercept** was positively associated with the **MyCogHealth** intercept (β=0.042, *p* = 0.01) and with improvement on MyCogHealth (β=0.011, *p* <0.05). No coupled changes were observed between **MyCogHealth** and **lifestyle** slopes (*p* = 0.25).

**Conclusion:**

The BHSP digital cognitive outcomes were similar to the NTB in their baseline associations with participant characteristics. The increased measurement frequency permitted by digital assessments revealed subtle improvements and cognition‐lifestyle associations not detectable by the NTB. These findings support digital assessments as flexible alternatives to traditional cognitive endpoints in lifestyle‐based dementia risk reduction studies.

